# Immunomodulatory Effects of Selected Non-Nutritive Bioactive Compounds and Their Role in Optimal Nutrition

**DOI:** 10.3390/cimb47020089

**Published:** 2025-01-31

**Authors:** Katarzyna Napiórkowska-Baran, Paweł Treichel, Anita Dardzińska, Agata Majcherczak, Anastazja Pilichowicz, Maciej Szota, Bartłomiej Szymczak, Ewa Alska, Justyna Przybyszewska, Zbigniew Bartuzi

**Affiliations:** 1 Department of Allergology, Clinical Immunology and Internal Diseases, Collegium Medicum Bydgoszcz, Nicolaus Copernicus University Torun, 85-067 Bydgoszcz, Poland; maciejszota98@gmail.com (M.S.); e.alska@icloud.com (E.A.); zbartuzi@cm.umk.pl (Z.B.); 2 Student Research Club of Clinical Immunology, Department of Allergology, Clinical Immunology and Internal Diseases, Collegium Medicum Bydgoszcz, Nicolaus Copernicus University Torun, 85-067 Bydgoszcz, Poland; treichel.pawel@gmail.com (P.T.); anita.dardzinska@op.pl (A.D.); agata.klaramajcheczak@gmail.com (A.M.); ana-pilichowicz@wp.pl (A.P.); bartlomiej.szymczak1@gmail.com (B.S.); 3 Department of Nutrition and Dietetics, Collegium Medicum Bydgoszcz, Nicolaus Copernicus University Torun, 85-067 Bydgoszcz, Poland; juprz@cm.umk.pl

**Keywords:** nutrition, immune system, omega-3 fatty acids, probiotics, prebiotics, postbiotics, vitamins, coenzyme Q10, sodium butyrate, microelements

## Abstract

The contemporary approach to nutrition increasingly considers the role of non-nutritive bioactive compounds in modulating the immune system and maintaining health. This article provides up-to-date insight into the immunomodulatory effects of selected bioactive compounds, including micro- and macronutrients, vitamins, as well as other health-promoting substances, such as omega-3 fatty acids, probiotics, prebiotics, postbiotics (including butyric acid and sodium butyrate), coenzyme Q10, lipoic acid, and plant-derived components such as phenolic acids, flavonoids, coumarins, alkaloids, polyacetylenes, saponins, carotenoids, and terpenoids. Micro- and macronutrients, such as zinc, selenium, magnesium, and iron, play a pivotal role in regulating the immune response and protecting against oxidative stress. Vitamins, especially vitamins C, D, E, and B, are vital for the optimal functioning of the immune system as they facilitate the production of cytokines, the differentiation of immunological cells, and the neutralization of free radicals, among other functions. Omega-3 fatty acids exhibit strong anti-inflammatory effects and enhance immune cell function. Probiotics, prebiotics, and postbiotics modulate the intestinal microbiota, thereby promoting the integrity of the intestinal barrier and communication between the microbiota and the immune system. Coenzyme Q10, renowned for its antioxidant attributes, participates in the protection of cells from oxidative stress and promotes energy processes essential for immune function. Sodium butyrate and lipoic acid exhibit anti-inflammatory effects and facilitate the regeneration of the intestinal epithelium, which is crucial for the maintenance of immune homeostasis. This article emphasizes the necessity of an integrative approach to optimal nutrition that considers not only nutritional but also non-nutritional bioactive compounds to provide adequate support for immune function. Without them, the immune system will never function properly, because it has been adapted to this in the course of evolution. The data presented in this article may serve as a foundation for further research into the potential applications of bioactive components in the prevention and treatment of diseases associated with immune dysfunction.

## 1. Introduction

Optimal nutrition is crucial for maintaining health and preventing various lifestyle-related diseases. In addition to macronutrients and essential vitamins, non-nutritive bioactive components have garnered significant attention for their impact on the human body at cellular and molecular levels. These include macro- and microelements and specific biologically active compounds that support immune function, influence metabolism, and aid in regenerative and anti-inflammatory processes.

This article presents the role of selected non-nutritive components in optimal nutrition. We will discuss the importance of macroelements such as magnesium, calcium, potassium, sodium, phosphorus, and sulfur, which contribute to electrolyte balance and nervous system functionality. We will also cover microelements—zinc, selenium, iron, iodine, copper, cobalt, chromium, manganese, molybdenum, and silicon—that play a vital role in numerous enzymatic reactions and protect against oxidative stress. Additionally, this article examines essential vitamins like vitamins D, E, C, and the B vitamins (B1, B6, B12, and folic acid), along with other bioactive immunomodulatory substances such as omega-3 fatty acids, prebiotics, postbiotics, probiotics, coenzyme Q10, alpha-lipoic acid and plant-derived components such as phenolic acids, flavonoids, coumarins, alkaloids, polyacetylenes, saponins, carotenoids, and terpenoids.

This article aims to underscore that, although often present in trace amounts, non-nutritive bioactive components play significant roles in maintaining health.

**Figure 1:**
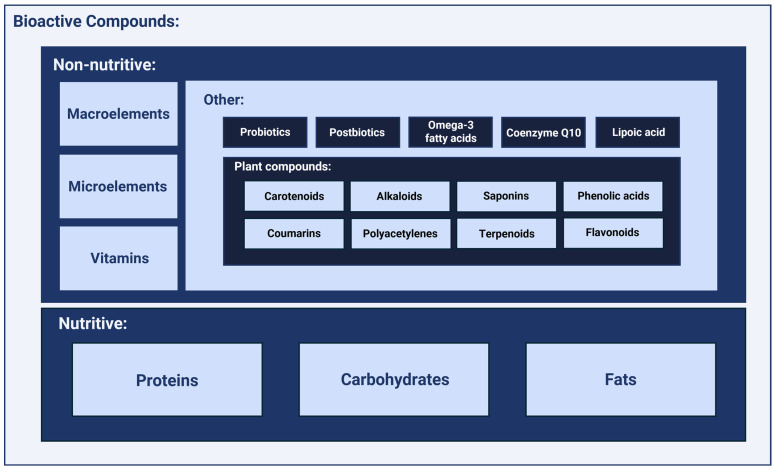
Divisions of bioactive compounds necessary for the proper functioning of the human body.

There are many divisions of bioactive compounds necessary for the proper functioning of the human body. The most accessible classification is presented in Figure 1.

## 2. Selected Non-Nutritive Bioactive Compounds and Their Role in Optimal Nutrition

### 2.1. Macroelements

Macronutrients are minerals essential for the proper functioning of the body and are found in larger quantities in tissues. They are needed in doses above 100 mg per day. Daily requirements and serum concentrations are presented in Table 1.

#### 2.1.1. Magnesium

Magnesium is a crucial macroelement in the human body, ranking as the fourth most abundant mineral. It is an essential cofactor in many biochemical processes, including DNA synthesis, protein production, energy metabolism, and glycolysis, participating in over 300 reactions within the human body [1,2]. Magnesium is also integral to muscle contraction, relaxation, and heart rhythm regulation due to its influence on transmembrane potassium and magnesium ion transport [2,3]. Serum magnesium concentrations below 0.85 mmol/L have been associated with increased disease risk [4,5]. Magnesium deficiency has been linked to a higher prevalence of arrhythmias, heart failure, and coronary artery disease, although the benefits of supplementation without a confirmed deficiency are inconclusive [6,7]. Magnesium deficiency has also been associated with type 2 diabetes, Alzheimer’s disease, and migraines [8,9]. Recommended daily intake for magnesium varies by age, gender, and health status [10]. Foods rich in magnesium include whole grains, nuts, potatoes, and leafy green vegetables [8,11].

Magnesium also plays a role in immune function regulation, serving as a cofactor in antibody production, facilitating proper binding to lymphocytes, and enabling macrophage response to lymphokines. It also takes part in the regulation of apoptosis. Magnesium deficiency increases oxidative stress in the body, potentially exacerbating inflammation. The effects of an overdose include diarrhea, nausea, muscle weakness, and heart rhythm disturbances [12,13].

#### 2.1.2. Calcium

Calcium is the most abundant macroelement in the human body, constituting approximately 2% of total body weight, with over 99% stored in bones [14,15]. Calcium’s primary role is to support proper skeletal mineralization and bone strength [16]. It also plays a part in various processes, including muscle contraction and cell apoptosis. It mediates cellular responses to hormonally active substances such as vasopressin, secretin, adrenaline, and glucagon [17,18,19]. Calcium has also been shown to activate protective reactions in cases of nervous system damage [20]. The primary dietary sources of calcium include dairy products, with 100 g of cheese containing approximately 1 g of calcium, and plant-based sources like kale and broccoli containing about 100–150 mg per 100 g [21,22,23,24].

Calcium also influences immune cell function, affecting their proliferation, differentiation, and gene transcription. Elevated calcium concentrations in mast cells stimulate degranulation and histamine release, while in macrophages, calcium increases the expression of pro-inflammatory cytokine genes. The effects of excessive substitution may include kidney stones, cardiac arrhythmias, or constipation [25,26].

#### 2.1.3. Potassium

Potassium is a primary intracellular electrolyte, with concentrations of approximately 1.6–2 g per kilogram of body weight in humans [27,28]. This macronutrient has numerous essential functions, from maintaining water–electrolyte balance to supporting muscle fiber function, nerve impulse transmission, and blood pressure regulation [27,29,30]. Studies indicate that 3–5 g of potassium intake per day may reduce type 2 diabetes risk and increase insulin sensitivity [31,32]. A potassium-rich diet may also lower cardiovascular risk by reducing reactive oxygen species production and increasing endothelial nitric oxide, which relaxes vascular endothelium and reduces blood pressure [32,33]. In a study of 10,341 participants, low serum potassium was associated with a higher prevalence of metabolic syndrome, increased uric acid and triglycerides, and lower concentrations of high-density lipoproteins [34]. The Dietary Guidelines Advisory Committee reports that foods with the highest potassium content per 100 g include tomato paste, Swiss chard, adzuki beans, Atlantic salmon, and potatoes, with concentrations of 1014 mg, 909 mg, 532 mg, 628 mg, and 544 mg, respectively [35,36].

Potassium also impacts immune function. As a primary component of intracellular fluid, potassium directly influences membrane potential, indirectly affecting cellular signaling pathways and intercellular communication. Pétrilli et al. demonstrated that low intracellular potassium concentration can activate the NALP3 inflammasome, increasing levels of pro-inflammatory interleukins IL-1beta and IL-18 [37]. High potassium concentrations in the tumor microenvironment can suppress effector T cell function and promote autophagy while maintaining stem-cell-like properties, resulting in CD8^+^ T cells with enhanced survival, multipotency, and anti-tumor abilities [38,39].

#### 2.1.4. Sodium

Sodium is the principal extracellular fluid electrolyte, with sodium cations and intracellular potassium primarily responsible for maintaining water–electrolyte balance [40,41]. Its other prominent roles include aiding nerve impulse transmission in nerve cells and muscle contraction and relaxation [40,42]. Sodium cations also transport various substances across the cell membrane via sodium-dependent cotransporters [43]. Na+/K+-ATPase, a major transmembrane protein that maintains sodium and potassium ion distribution, can also serve as a receptor and participate in cellular signaling [44].

The World Health Organization recommends limiting daily sodium intake to 2000 mg for adults, equivalent to less than 5 g of table salt. However, adults’ average daily sodium intake is 4310 mg, with excess sodium intake estimated to cause over 1.89 million deaths annually [45,46,47]. Sodium is found in many foods, mainly processed items like meats, snacks, dairy products, breads, and seasonings such as fish sauce and soy sauce [46,48].

Sodium also plays a role in immune function [49]. Müller et al. found that high sodium concentrations in interstitial fluid can promote chemotaxis and macrophage migration to areas of increased sodium [50]. Additionally, osmotic stress from hypertonic sodium chloride solutions fosters the proliferation of human macrophages and mononuclear cells while enhancing cytokine production [49,51,52]. High sodium intake is associated with an increased risk of hypertension, partly through its pro-inflammatory effects [53]. A high-sodium diet also disrupts gut microbiota, which can lead to metabolic and autoinflammatory diseases [49,54,55,56].

#### 2.1.5. Phosphorus

Phosphorus is the second most abundant mineral in the human body and the sixth most abundant element [57,58]. It plays essential roles as a component of DNA, RNA, various phosphoproteins, and ATP, the leading cellular energy carrier [59,60]. Phosphorus also contributes to cell signaling as cyclic AMP and participates in cell membrane formation as glycerophospholipids [60]. Phosphorus-rich foods include dairy, meat, fish, whole grains, and processed foods with phosphorus additives to enhance appearance and texture [61,62,63,64]. Phosphorus intake often exceeds the recommended daily amount due to additives in processed foods [63,65]. Excess phosphorus intake has been shown to increase the risk of chronic kidney disease and lead to the pathological calcification of tissues, subsequently elevating the risk of cancer, hypertension, cardiovascular diseases, and dementia [57,63,65].

Yang et al. demonstrated that hyperphosphatemia could shorten telomere length in leukocytes, leading to accelerated cellular aging [66]. Kuro et al. observed that elevated phosphorus concentrations may exert toxic effects and accelerate aging throughout the body [67].

#### 2.1.6. Sulfur

Sulfur is a nonmetallic element that ranks seventh in abundance among elements in the human body [68]. It can be supplied as inorganic sulfates or organic sulfur compounds [69].

Sulfur plays a critical role in the human body as a cofactor for enzymes that regulate oxidative reactions [68]. It is also a key component of two amino acids—methionine and cysteine—essential for the proper structure of human proteins such as keratin [69]. Additionally, sulfur is a vital component of 3′-phosphoadenosine-5′-phosphosulfate, which is involved in synthesizing heparin, insulin, and chondroitin, a principal constituent of bones and cartilage [70,71]. Sulfur is also present in glutathione, which fulfills multiple crucial functions in the human body. It acts as a cofactor for antioxidant enzymes, neutralizes reactive oxygen species, regulates cell proliferation and apoptosis, and influences inflammatory processes by modulating prostaglandin synthesis [70,72,73]. Studies have shown that sulfur deficiency, which may affect individuals on a vegetarian diet, can increase the risk of cardiovascular diseases and stroke [68]. Furthermore, the average intake of sulfur-containing amino acids among adult Americans, primarily from animal-derived products, exceeds the recommended daily intake by more than 2.5 times, a factor associated with an increased risk of cardiometabolic diseases [74].

The concentration of sulfur in human serum is approximately 1200 mg/L [75]. In the human body, sulfur is primarily supplied as methionine, an essential amino acid, and cysteine, a non-essential amino acid. Small amounts of sulfur are also found in foods such as broccoli, garlic, and onions [69,72]. The estimated daily requirement for inorganic sulfur is around 250 mg, typically met through the diet [68,72]. The recommended daily intake for methionine (combined with cysteine) is 14 mg/kg of body weight. It is worth noting that animal-based products such as fish, beef, and poultry contain approximately 5% sulfur-containing amino acids, whereas plant-based foods contain less than 4% [72,76].

### 2.2. Microelements

Micronutrients, also referred to as trace elements, are minerals that are essential to the body in very small amounts, usually less than 100 mg per day. Despite the minimal demand for them, these elements exert considerable influence over a multitude of biological processes, including metabolism, enzyme function, hormonal regulation, and other vital biological activities. Daily requirements and serum concentrations for known microelements are presented in Table 1.

#### 2.2.1. Zinc

Zinc is an essential trace element, crucial for many processes in the human body. As a regulator and coenzyme, it supports the function of many enzymes, participates in the synthesis of DNA, RNA, and proteins, and acts as an antioxidant. Zinc stabilizes cell membranes, supports the functioning of the immune system, and regulates the production of hormones and their receptors [77]. In the blood, zinc is predominantly bound to albumin. The immune system is very sensitive to changes in zinc concentration, which affect both specific and non-specific responses. Zinc plays a key role in cytokine production and processes such as chemotaxis, phagocytosis, and the destruction of pathogens by reactive oxygen species. Zinc deficiency impairs the function of granulocytes, natural killer (NK) cells, and macrophages, increasing the production of pro-inflammatory cytokines such as IL-1β, IL-6, and TNF-α. Zinc is also essential for proper antigen recognition by T cells, and its absence impairs NK cell cytotoxicity by interfering with the recognition of a set of proteins responsible for antigen presentation to T cells by NK receptors p58. Zinc stimulates CD8^+^ lymphocyte proliferation in cooperation with IL-2 and increases leukocyte adhesion to the endothelium, which promotes their activation. It also promotes the cytolytic activity of T lymphocytes, which eliminate pathogens and tumor cells by recognizing them through the interaction of an antigen-recognizing receptor on T lymphocytes with an oligopeptide presented by major tissue compatibility system molecules [78].

A study conducted by Jothimani et al. in which both male and female patients between the ages of 18 and 77 were included in the study and control groups clearly showed that 49 patients infected with COVID-19 had significantly lower concentrations of zinc in their bodies compared to healthy subjects [79]. Importantly, a clear correlation was also noted between low baseline zinc levels and a worse disease course. Patients deficient in this element were more likely to experience complications that led to prolonged hospitalization. Moreover, low zinc concentrations were also associated with an increased risk of death, suggesting that adequate zinc concentration may play a key role in the prognosis and overall course of the disease in coronavirus-infected individuals. In turn, high concentrations of zinc may induce nausea, vomiting, weakened immunity, and impaired copper absorption [79].

#### 2.2.2. Selenium

Selenium is a vital element in maintaining equilibrium within the human body. About 50% of selenium is found in skeletal muscles. It acts as an antioxidant and is a component of selenoproteins, such as glutathione peroxidase, which protects the thyroid from excess hydrogen peroxide. Selenoproteins facilitate optimal thyroid functionality and modulate the body’s inflammatory and immune responses [77]. Adequate selenium concentrations strengthen the immune system, enhancing its capability to subdue viral pathogens and regulate both autoimmune and inflammatory responses. Selenium supplementation is mainly beneficial in cases of selenium deficiency [77].

As Sadler et al. point out in their paper, selenium is essential for both innate and acquired immunity, stimulating the activity of immune cells such as macrophages and T lymphocytes [80]. Selenium may also affect the body’s ability to counteract viral infections. Selenium deficiency can increase the virulence of viruses, and adequate concentrations of selenium in the diet are crucial, as low concentrations can increase susceptibility to infection, while excess levels can be toxic and lead to hair loss, brittle nails, gastrointestinal disorders, and neurological changes. There are multiple guidelines for daily selenium intake, although further research is required to refine them in order to ensure sufficient amounts without risk of toxicity. The immunomodulatory potential of selenium, obtained from both diet and supplementation, may be important in preventing infections, autoimmune diseases, and promoting recovery in chronic diseases [80].

A study by Hu Y et al. that included patients with Hashimoto’s disease and healthy controls showed that selenium treatment significantly reduced the concentration of thyroid peroxidase antibodies and thyroglobulin antibodies [81]. A decrease in thyrotropic hormone levels was also observed. Se supplementation led to an increase in concentrations of selenium, glutathione peroxidase 3, and selenoprotein P1 compared to the control group. There was also an increase in the number of activated regulatory T cells and their subsets, suggesting that Se may promote immunoregulatory mechanisms associated with thyroid autoimmunity [81].

#### 2.2.3. Iron

Iron is an essential mineral that supports principal body functions such as red blood cell production, cell metabolism, and proper immune system function. Iron plays an important role in immunity—iron deficiency impairs the function of T and B lymphocytes, which can be improved with supplementation. However, excess iron can increase the risk of infections, such as malaria, as pathogens use iron for their development [77]. Iron is crucial for B cell maturation and differentiation. Iron ions in the trivalent form, bound to transferrin, are reduced to the divalent form by the enzyme STEAP3, allowing their transport into the cytoplasm. Iron deficiency leads to the disruption of B cell activation and proliferation, as the iron-dependent demethylation of histone 3 lysine 9 regulates the expression of genes, such as the cyclin E1 gene, which is necessary for B cells to enter the S phase of the cell cycle. As a result, iron deficiency results in impaired antibody responses to antigens, including responses to vaccination, highlighting the importance of this element for humoral immunity [82]. In the course of an acute infection, the host body reduces the availability of iron, which inhibits the development of pathogens. In contrast, with long-term activation of the immune system, caused by chronic infection, autoimmune disease, or cancer, iron is retained not only from microorganisms, autoreactive lymphocytes, and tumor cells but also from erythroid precursor cells. This mechanism is one of the main factors contributing to the development of anemia in chronic inflammation [83].

A study conducted at the Center for Disease Control and Prevention in Zhejiang Province analyzed the relationship between iron metabolism and the humoral immune response to the measles virus. In a group of 118 participants aged 10+ years, it was found that those with a low iron concentration (<50 µg/dL) had reduced titers of measles-virus-specific IgG antibodies than those with normal iron levels. In addition, transferrin saturation (TS < 16%) correlated with a lower immune response. Other factors tested, such as vitamin D, had no significant effect on the antibody response, indicating a key role for iron in the humoral response [82,84]. In contrast, a study by Polak et al. analyzed the effects of the consumption and supplementation of foods rich in iron, among other things, on the frequency and course of infections. Regular iron intake was associated with a lower frequency of viral infections and occasional intake correlated with its milder course. Iron overdose can cause damage to the liver, pancreas, and heart, as well as constipation and nausea [85].

#### 2.2.4. Iodine

Iodine is an element needed for the synthesis of thyroid hormones, which regulate the body’s metabolic functions. Iodine enters the thyroid cells via the sodium iodide symporter and is then transported by the PENDRIN receptor, allowing it to be converted into T4 and T3 hormones, which are crucial for regulating metabolism [86]. Thyroid hormones also affect immunity, increasing anti-tumor cell activity and cytokine production. Interestingly, iodine may have a direct effect on immune cells, although the mechanisms are still under investigation.

A study by Cuenca-Micó et al. analyzed the effects of molecular iodine on the immune microenvironment of breast cancer tumors. The administration of iodine has been demonstrated to stimulate anticancer immune pathways at different stages of tumor development [87]. In comparison to the control samples, I_2_ stimulated Th1- and Th17-type responses, enhancing NK cell cytotoxicity, T and B receptor activity, and antigen presentation processes. A deconvolution analysis revealed an initial augmentation in the number of macrophages and dendritic cells, followed by an increase in CD4⁺ T and B cells in response to chemotherapy and iodine. The analysis of cytotoxic markers confirmed an increase in the anti-tumor response induced by I_2_ accompanied by a decrease in oncogenic markers such as transforming growth factor (TGF) β. The evaluation of interferon (IFN)γ and TGFβ gene promoter methylation showed that I_2_, especially in combination with chemotherapy, promoted the activation of anti-tumor genes IFNγ and silenced pro-oncogenic genes TGFβ. Excess iodine can cause thyrotoxicosis, but also a metallic taste in the mouth [87].

#### 2.2.5. Copper

Copper is a trace element that is essential to human health in small amounts; yet, it can be toxic in excess. In the human body, copper acts as a cofactor in a variety of respiratory and metabolic processes, as well as in the regulation of oxidative balance. The majority of this amount is stored in the liver, bones, and muscles. Excess copper is processed by the liver and excreted from the body via bile. Copper exerts a pronounced effect on the immune system as its deficiencies lead to a deterioration in immune function. It has been demonstrated that elevated copper concentrations in the bloodstream can enhance immune function. However, excessive copper intake is also utilized by certain pathogens, indicating the necessity for an optimal balance of Cu within the body. Nutritional intake influences copper concentration, with intakes below 0.8 mg per day resulting in copper loss and intakes above 2.4 mg per day leading to increased Cu concentration [77]. Copper serves as a signal regulator of alpha-kinase 1 (ALPK1) kinase activity—a receptor responsible for molecular pattern recognition. In response to infection, host cells accumulate copper in the cytosol, thereby enhancing their defense against pathogens, both intracellular and extracellular. Copper has been demonstrated to activate the ALPK1-dependent innate immune pathway through direct binding to ALPK1, thus increasing its sensitivity to ADP-heptose, a bacterial metabolite. Consequently, host cells elicit a stronger immune response to the infection. It has also been discovered that the presence of copper augments the production of pro-inflammatory cytokines, mobilizes phagocytic cells, and promotes bacterial elimination. Effects of overdose may include liver damage, nausea, diarrhea, and neurological disorders [88].

#### 2.2.6. Cobalt

Cobalt is an essential element for human and animal metabolism, primarily due to its role in vitamin B12, in the structure of which the cobalt ion is situated in the center and stabilized by four nitrogen atoms. Vitamin B12 is mainly found in animal products and is involved in the conversion of carbohydrates into energy, as well as in the methionine cycle, where it facilitates homocysteine metabolism. Cobalt deficiency can result in the development of anemia, thyroid disorders, and congenital abnormalities [89]. Conversely, while less prevalent, excess cobalt has been observed to stimulate the production of red blood cells. For this reason, cobalt salts are occasionally employed in sports medicine to enhance performance. Excessive exposure to cobalt has been linked to the development of DNA damage and oxidative stress, which in turn increases the risk of developing lung cancer and thyroid disorders. Cobalt can be absorbed into the body via the gastrointestinal tract, the respiratory system, or the skin, making it a valuable element for use in a wide range of industries and medicine [90].

A study conducted by Lin et al. demonstrated that magnetic nickel–cobalt alloy nanocrystals inhibit the activation of inflammasomes, receptor complexes within the immune system that initiate inflammatory responses [90]. Furthermore, the nanocrystals were observed to diminish neutrophil recruitment in a murine model of acute peritonitis and mitigate symptoms of colitis by inhibiting the activation of inflammasomes. The findings of the study suggest that nickel- and cobalt-based nanomaterials may have the potential to be utilized in the development of anti-inflammatory nanomedicine therapies targeting macrophage-dependent inflammatory diseases [91].

#### 2.2.7. Chromium

Chromium is predominantly present in two forms: hexavalent and trivalent. The hexavalent form of chromium has been identified as a carcinogen for humans, whereas the trivalent form, which exhibits biological activity, is an essential component of metalloenzymes. The most common dietary sources of chromium are whole grains, broccoli, grapes, potatoes, garlic, basil, beef, oranges, apples, bananas, turkey, mushrooms, and green beans [92].

The third oxidation state of chromium is well tolerated, and no serious adverse events have been observed in individuals taking dietary supplements containing chromium in the form of chromium picolinate, chromium chloride, chromium nicotinate, chromium polynicotinate, chromium citrate, chromium histidinate, and high chromium yeast, so a tolerable upper intake level has not been established [91]. Nevertheless, caution is advised due to the paucity of scientific data on the subject. Chromium is stored in the liver, spleen, soft tissues, and bones, and in vivo, it enhances insulin action in peripheral tissues and is involved in carbohydrate, protein, and fat metabolism and oxidation [93].

The precise role of chromium in the immune system remains unclear. A number of studies have proposed the potential of chromium supplementation to reduce inflammatory markers in the bloodstream and oxidative stress at the cellular level. However, the findings remain inconclusive. Some studies have corroborated the hypothesis that chromium may potentially exert an influence on inflammatory and immune system processes. The supplementation of chromium affects the total antioxidant capacity of the body and lowers the concentration of malondialdehyde (MDA), which is a marker of oxidative stress and a product of lipid peroxidation by free radicals. MDA has a proven atherogenic effect. In addition, MDA covalently interacts with proteins and nucleic acids in mitochondria, resulting in cross-links that cause damage. Various epitopes of MDA interact with the innate immune system, mainly by affecting the increased expression of pro-inflammatory genes and the activation of downstream inflammatory signaling pathways, including protein C, p38-MAPK, ERK1/2, and NF-kB. In addition, lipid peroxidation is implicated in the development of oxidative stress, which has a profound impact on many processes, including the immune system. Numerous studies have shown a reciprocal amplification between oxidative stress and inflammation in allergic airway diseases. Therefore, reducing MDA concentrations by chromium supplementation has an impact on the immune system [92]. Additionally, a reduction in inflammatory biomarkers, such as hs-CRP or TNF-α, was noted [94,95]. Due to the small number of studies that evaluate the effect of chromium supplementation on the immune system, for this reason, more research is required to determine its effect with accuracy.

#### 2.2.8. Manganese

Manganese plays a vital role in numerous physiological processes and is present primarily in two oxidation states: Mn2+ and Mn3+. The predominant form of manganese present in tissues and blood is in the second oxidation state. In the human body, manganese is mainly present in the bones, liver, kidneys, pancreas, and adrenal and pituitary glands. In the body, manganese participates in a number of processes, including the regulation of blood sugar and cellular energy, reproduction, digestion, bone growth, blood clotting and hemostasis, antioxidant defense, and normal immune function. Manganese plays a crucial role in the function of metalloproteinases, which encompass a diverse range of enzymes including oxidoreductases, transferases, hydrolases, lyases, isomerases, and ligases [94].

The precise role of manganese in the immune system remains to be fully elucidated. Multiple studies have demonstrated a significant effect of the element on the immune system’s response to infection. The effects of manganese supplementation have been observed to be both positive and negative. It has been demonstrated that the exposure of human monocytes or macrophages to manganese results in an increase in the production of cytokines, including IL-1β, IL-6, IL-8, IFN-γ, and TNF-α, which indicates that manganese has immunomodulatory functions [96]. Moreover, elevated concentrations of manganese have been demonstrated to influence immune cells, including the chemotaxis and motility of neutrophils, which are enhanced by increased adhesion to various extracellular matrices [97,98]. Additionally, the impact of manganese on the maturation of dendritic cells and macrophages has been documented. Manganese ions (Mn2+) have been demonstrated to influence the presentation of specific antigens by dendritic cells, as well as to enhance the differentiation of CD8^+^ T cells. Furthermore, the same study investigated the impact of elevated manganese concentrations on NK cell activation and the expansion of CD8^+^ memory T cells [99]. It should be noted that elevated manganese concentrations are not the only factor affecting the immune system. Lower concentrations of this element in the body have been observed to affect the abnormality of antibody production and secretion. It is pertinent to note that, due to the prevalence of manganese in the general environment and in typical dietary sources, manganese deficiency is a relatively uncommon occurrence in humans [100].

Interestingly, manganese was found to exert a direct influence on the immune system during the system’s response to specific infections, as Mn2+ was identified as a crucial element in host defense against DNA viruses. The release of Mn2+ from membrane-enclosed organelles during viral infection resulted in its accumulation in the cytosol, where it increased the sensitivity of double-stranded DNA by acting on the DNA sensor cGAS (cyclic GMP-AMP (cGAMP) synthase) [101]. This finding substantiates the assertion that manganese is a critical component of host defense against DNA viruses.

The other discovered immune mechanism in which manganese participates is the modification of the activity of manganese superoxide dismutase (MnSOD), an enzyme located in mitochondria, which protects the mitochondria from oxidative damage. The enzyme has been employed in research as a prospective anti-inflammatory agent on account of its capacity to scavenge superoxide anions, which exert a pro-inflammatory influence by instigating lipid peroxidation and oxidation, DNA damage, peroxynitrite ion formation, and neutrophil recruitment to sites of inflammation [102].

While there is a substantial body of evidence indicating that manganese has beneficial effects on immune function, it is imperative to also consider the toxic properties of manganese, which are involved in the conversion of hydrogen peroxide into oxygen radicals through the Fenton reaction. Accordingly, elevated concentrations of manganese can induce cellular toxicity and may contribute to oxidative stress and genetic material damage. Additionally, evidence indicates that high concentrations of manganese can impede the functioning of immune cells. Elevated concentrations of this element have been demonstrated to accelerate primary degranulation while simultaneously impairing the suicidal formation of neutrophil extracellular traps, which ultimately results in a reduction in bactericidal activity [103]. Additionally, manganese has been demonstrated to exert detrimental effects on the lymphoid cell lineage [104].

### 2.3. Vitamins

Vitamins are a group of organic chemical compounds of various structures, necessary for the proper functioning of a living organism, although they are not a source of energy for it or a building block for cells. The human body can synthesize only a few vitamins (e.g., vitamin D, K, B3, and biotin), and in most cases, it is necessary to provide vitamins via one’s diet or supplementation to cover the full demand. Daily requirements and serum concentrations for vitamins are presented in Table 1.

#### 2.3.1. Vitamin D

Vitamin D fulfills a multitude of functions within the human body. It exists in multiple forms, including calciferol (vitamin D1), ergocalciferol (vitamin D2), and cholecalciferol (vitamin D3). It plays a significant role in the regulation of calcium and phosphate metabolism. It stimulates the intestinal absorption of calcium and phosphate, regulates bone metabolism, and exerts negative control over the secretion of parathormone (PTH) through the hormonal action of the active metabolite calcitriol [104]. Despite the presence of vitamin D in food sources such as fatty fish, eggs, and certain types of mushrooms, these dietary sources are unable to meet the body’s demand for this essential nutrient. Additionally, vitamin D is endogenously produced in the skin from cholesterol in response to UV-B radiation [94].

In the liver, cholecalciferol is hydroxylated to 25-hydroxyvitamin D and subsequently converted to the active hormone 1,25-hydroxyvitamin D (1,25(OH)2D) in the kidneys. It is solely the active form, 1,25(OH)2D, that exerts its effects on VDRs [105].

Vitamin D exerts its function intracellularly through transcriptional effects on the genome via the receptor for vitamin D (VDR) within the cell nucleus and through non-genomic effects, whereby the VDR induces rapid signaling localized to the cell membrane and/or cytoplasm [106]. The vitamin D receptor is expressed in a multitude of tissues throughout the body, including the skin, immune system, bones, and endocrine organs [93]. Vitamin D has been demonstrated to elicit a range of immunomodulatory, anti-inflammatory, antioxidant, and antifibrotic effects on numerous tissues [107].

The impact of vitamin D on the immune system is intricate and contingent upon a multitude of pathways. One pathway of the immunomodulatory action of vitamin D is the local production of calcitriol by immune cells. In this manner, it exerts an autocrine or paracrine immunomodulatory effect. Additionally, immune cells that produce calcitriol contain the vitamin D receptor (VDR) and the requisite enzymes for metabolizing vitamin D3. Moreover, direct and indirect immunomodulatory effects on T lymphocytes, B lymphocytes, and antigen-presenting cells (dendritic cells and macrophages) have been demonstrated, affecting both innate and adaptive immune responses [108]. Additionally, research has shown that vitamin D enhances immunity, providing protection against pathogens, while on the other hand exerting immunosuppressive effects, preventing the deleterious effects of prolonged inflammatory responses on the host [106].

Vitamin D also provides immunomodulatory effects by enhancing innate cellular immunity, which is achieved by stimulating the expression of antimicrobial peptides, including cathelicidin and defensins. Defensins maintain the structure and integrity of tight and gap junctions while also enhancing the expression of antioxidant genes. Vitamin D is known to maintain the integrity of these connections [109]. In the presence of active vitamin D, the stimulation of CD4^+^CD25^−^T cells was observed, resulting in the inhibition of the production of pro-inflammatory cytokines, including IFN-gamma, IL-17, and IL-21. The effect of vitamin D on T cells causes them to act in conjunction with IL-2 as potent anti-inflammatory agents and physiological inducers of adaptive regulatory T cells. Additionally, it influences the differentiation of monocytes into macrophages while enhancing superoxide production, phagocytosis, and bacterial destruction [109].

Given its extensive impact on the immune system, vitamin D supplementation is advised in instances where its immunomodulatory effects are desired. Some studies have indicated that supplementation represents a crucial element in public health initiatives and may prove beneficial in the treatment of dysimmune diseases. A study examining the impact of vitamin supplementation during the course of a SARS-CoV-2 infection revealed that vitamin D supplementation may have the potential to reduce the concentration of pro-inflammatory cytokines, thereby reducing mortality rates associated with acute respiratory distress syndrome in patients with SARS-CoV-2 infection. The effects of an overdose may include kidney stones, muscle weakness, or heart problems [105].

In the case of the effect of vitamin D supplementation on the immune system, many studies have been produced in a wide variety of groups and also in the course of diseases such as SARS-CoV-2. This gives us a very rich picture of the effect of this vitamin on the body. However, further studies of the effects of vitamin D supplementation on the course of other diseases need to be conducted.

#### 2.3.2. Vitamin E

In the human body, vitamin E, in its α-tocopherol form, is the most biologically active. It is a component of all biological membranes and is the most important fat-soluble antioxidant, protecting membrane lipids and lipoproteins from lipid peroxidation [94]. The supplementation of vitamin E in the diet of humans is restricted to alpha-tocopherol due to the biological activity of this form and the physiological characteristics of the human body. Other forms of vitamin E are poorly recognized by hepatic alpha-tocopherol transfer protein (TTP) and are not converted to alpha-tocopherol by humans, rendering them unusable in the human body [110].

Due to its antioxidant properties, vitamin E has a profound effect on the functioning of the immune system. One of the mechanisms through which α-tocopherol affects the immune system is by altering the level of oxidative stress. It is defined as an imbalance between the amount of reactive oxygen species and the comparatively low antioxidant capacity of the biological system [93]. The accumulation of oxygen radicals then occurs, and in the presence of vitamin E, superoxide radicals react with α-tocopherol in place of lipid hydroperoxide. The chain reaction of superoxide radical generation is terminated, and further fatty acid oxidation in the membrane is prevented [111].

By virtue of its presence in the plasma membrane, vitamin E affects the interaction of membrane proteins and the translocation of enzymes to the plasma membrane, thereby modulating the activity of signal transduction enzymes [112]. Consequently, it is capable of influencing a number of enzyme pathways, including protein kinase C (PKC) activity. Vitamin E exerts its inhibitory effect on PKC activity by activating protein phosphatase and phosphorylating the enzyme. The inhibition of PKC has been observed in immune cells, including monocytes, macrophages, and neutrophils. Consequently, the proliferation of these cells is diminished, and the production of superoxide in neutrophils and macrophages is reduced. Furthermore, numerous studies have demonstrated that vitamin E supplementation is associated with an increased delayed-type hypersensitivity response, increased IL-2 production, and decreased IL-6 production [111,112,113,114,115].

It has been observed that immune cells are particularly rich in vitamin E, presumably to protect the high membrane content of polyunsaturated fats from oxidative damage resulting from their high metabolic activity and their normal defense function [116]. Thus, vitamin E supplementation has been shown to affect the relationship between immune cell activity and α-tocopherol concentration in the body. The impact of vitamin E supplementation on macrophages by inhibiting the activity of nuclear factors of alveolar macrophages erythroid 2-related 2 (NRF2) following allergen exposure has been documented. This suggests the potential for vitamin E to exert a protective effect in allergies and asthma [111]. The impact of vitamin E supplementation on NK cell cytotoxicity was also examined. In a study involving patients who received two weeks of supplementation, 750 mg of vitamin E in patients with colorectal cancer resulted in an increase in NK cell activity in six of the seven patients. Although short-term vitamin E supplementation was observed to affect NK cells, long-term treatment did not result in alterations in perforin expression or IFN-γ production, which were of particular interest in this study. Consequently, the mechanisms underlying the enhancement of NK activity by vitamin E remain unclear [117].

It is also noteworthy that vitamin E supplementation has been demonstrated to affect the humoral response, resulting in enhanced antibody production [115]. Additionally, in vitro vitamin E supplementations of primary human T cells have been observed to inhibit CD95L expression and cell death [118].

Vitamin E indirectly regulates T cell activity through the modulation of inflammatory mediators, including pro-inflammatory cytokines and prostaglandin E2, which ultimately suppress T cell responses. Prostaglandin E2 exerts its influence on both the innate and adaptive immune systems through the inhibition of T cell proliferation, IL-2 receptor expression, and IL-2 production. Effects of an overdose may include blood clotting disorders, increased risk of bleeding, and muscle weakness [119].

The precise immunomodulatory effect of vitamin E supplementation remains uncertain. A range of mechanisms of action have been observed for α-tocopherol, indicating the potential for a protective effect on hypersensitivity reactions and the promotion of antibody production by cells. It has been unequivocally demonstrated that vitamin E supplementation exerts a significant influence on immune system function.

#### 2.3.3. Vitamin C

Vitamin C, also known as ascorbic acid, is a water-soluble vitamin found in human cells and plasma [120]. Foods that are rich in vitamin C are widely available. The majority of the daily intake is derived from fruits, vegetables, potatoes, and juices. The richest sources of vitamin C are the Kakadu plum, camu-camu, acerola, rosehips, and sea buckthorn fruits, which are utilized in the production of supplements. Vitamin C is primarily recognized for its antioxidant properties; nevertheless, elevated dosages have been demonstrated to act as a pro-oxidant. Vitamin C plays a role in the synthesis of collagen, hormones, and carnitine. Furthermore, it regulates gene transcription and translation. Vitamin C has been demonstrated to enhance iron absorption, which is why it is often added to oral preparations containing iron [121].

Vitamin C, in addition to its role in metabolic functions, helps boost immunity and fight infections by enhancing both innate and adaptive immune responses. The immune-boosting influence is likely associated with its role as a cofactor for several gene regulatory and biosynthetic enzymes. The accumulation of vitamin C in neutrophils augments chemotaxis and phagocytosis, and as a result, it increases microbial killing. Moreover, vitamin C promotes the clearance of used neutrophils from the site of infection [122]. The action of vitamin C also results in reduced neutrophil necrosis [123]. In vitro studies showed the inhibitory effect of vitamin C on the expression of pro-inflammatory mediators such as interleukin-6 (IL-6) and tumor necrosis factor alpha (TNF-α) in adult whole blood cells. This fact suggests that vitamin C may prevent an excessive immune response in patients susceptible to the development of systemic inflammatory response syndrome, although further studies are needed. Vitamin C appears to influence pro-inflammatory processes through altering lipopolysaccharide-induced gene expression in human macrophages via the nuclear factor kappa-light-chain-enhancer of activated B cells (NF-κB). A clinical study involving healthy male students showed that vitamin C increases the concentration of IgA and IgM in blood. Furthermore, according to research results, the in vitro administration of vitamin C increases the ability of NK cells to kill tumor cells. Leukocytes accumulate vitamin C in the presence of a concentration gradient, indicating the importance of vitamin C in the functioning of immune cells. Additionally, vitamin C limits the development of several bacterial species and inhibits the growth of other bacterial species [124]. A randomized clinical trial conducted in 200 patients showed that adding a supplementation of vitamins C and E to standard triple therapy increases the eradication rate of Helicobacter pylori [122]. Vitamin C is viewed as an antiviral agent because it enhances the concentration of antiviral cytokines, such as interferon (IFN)-α/β. Moreover, it increases free radical formation to decrease viral yield [124]. During viral infections, vitamin C concentration decreases. Deficiency of vitamin C seems to play a role in the development of postherpetic neuralgia. A multicenter prospective cohort study showed that in 67 patients suffering from symptomatic herpes zoster, the intravenous administration of vitamin C (Pascorbin^®^ 7.5 g/50 mL) for two weeks in combination with standard therapy reduced the risk of developing postherpetic neuralgia. To confirm these findings, randomized, placebo-controlled clinical studies are needed [122]. Vitamin C deficiency impacts immune functions—it increases oxidative damage, decreases delayed-type hypersensitivity responses, and impairs the wound-healing process. Moreover, it is associated with a higher risk and severity of pneumonia and other infections. Potential side effects of excess vitamin C include oxalate nephropathy, prooxidant activity, and false-positive hyperglycemia in glucose measurements taken with a glucometer. Vitamin C accelerates the elimination of drugs by the kidneys (increases the rate of elimination of tricyclic antidepressant derivatives), reduces the effectiveness of phenothiazine derivatives and aminoglycosides, and increases the concentration of exogenous estrogens in serum [125].

#### 2.3.4. Vitamin B1

Vitamin B1, commonly referred to as thiamine or thiamin, is a water-soluble vitamin that presents multidirectional biological activity. It serves as a coenzyme that is essential for the metabolism of carbohydrates, fats, and proteins. It participates in cellular respiration, fatty acid oxidation, mitochondrial energy production, and protein synthesis. Moreover, vitamin B1 exhibits robust antioxidant properties. Thiamine is indispensable for the optimal functioning of the central and peripheral nervous systems, where it fulfills both enzymatic and non-enzymatic roles. It facilitates the synthesis of neurotransmitters [126].

Moreover, it participates in the process of myelinogenesis, which in turn affects the speed of nerve conduction. Vitamin B1 has been demonstrated to inhibit the activity of acetylcholinesterase. Additionally, it is a constituent of the axoplasmic, mitochondrial, and synaptosomal membranes. Some studies have demonstrated that thiamine can play a role in preventing depression. The recommended daily intake of vitamin B1 depends on age, sex, and physiological condition. This vitamin is present in various food sources, including enriched bread, whole grain cereals, peas, beans, nuts, brown rice, pork loin, pork shoulder, and beef [127].

In addition to its metabolic and structural functions, vitamin B1 has also been demonstrated to possess immunomodulatory properties. The interactions between vitamin B1 and immune cells are mediated by heme-dependent oxygenases, which affect the release of intracellular adhesion molecules that are used to localize cells expressing a variety of integrins. Due to its antioxidant properties, thiamine protects cell surface sulfhydryl groups located on neutrophils from free radical damage. Furthermore, in macrophages, vitamin B1 has been demonstrated to inhibit processes induced by oxidative stress, including the activation of NF-κB and the release of pro-inflammatory cytokines [128]. Given its antioxidant properties, vitamin B1 exhibits anti-inflammatory and anti-tumor properties, as it enhances the phagocytic activity of macrophages [127]. A deficiency in vitamin B1 has been linked to the onset of neuroinflammation, T cell infiltration, an overproduction of pro-inflammatory cytokines, and an increased expression of CD40 and CD40L by microglia and astrocytes [124]. Vitamin B1 deficiency is also associated with Alzheimer’s disease. One of the reasons may be the fact that in brain regions where the activity of thiamine-dependent enzymes is reduced, neuronal death occurs [128]. Vitamin B1 suppresses microglial cells’ prooxidative activity, and that is why it has the potential to be used in treating neurodegenerative diseases—further studies are needed [120]. An important issue regarding thiamine supplementation is its oncogenic potential, which remains unknown. Additional studies are required to distinguish vitamin’s B1 beneficial effects in healthy patients distinctly from its effect in patients with cancer, where it may be therapeutic or contribute to tumorigenesis. The results of research to date are mixed and do not provide clear answers. Overdoses of vitamin B1 (thiamine) are very rare because it is a water-soluble vitamin and its excess is usually excreted in the urine. However, in exceptional situations, especially with excessive supplementation or intravenous administration, some side effects may occur, such as gastrointestinal disturbances, dizziness and headaches, excessive sweating and feelings of heat, or tachycardia [128].

#### 2.3.5. Vitamin B6

The significance of vitamin B6 within the human body is exemplified by its function as a coenzyme in over 150 distinct biochemical reactions. It plays a role in the metabolism of carbohydrates, lipids, amino acids, and nucleic acids. Moreover, it participates in cellular signaling pathways. Vitamin B1 also demonstrates antioxidant properties. Moreover, it plays a role in the regulation of blood pressure and the coagulation process [129].

Vitamin B6 is an essential component of the immune system, facilitating its optimal functioning. It plays a role in both innate and acquired immunity. It takes part in the production of T lymphocytes and interleukins. Additionally, it elevates the concentration of the cytokine IL-10, which exhibits anti-inflammatory and immunosuppressive properties [130]. Studies have shown *that it* exerts an influence on the activity of inflammasomes, thus reducing inflammation. A deficiency in vitamin B6 has been linked to a reduction in antibody production, a decline in IL-2 concentration, and an increase in IL-4 concentration. Additionally, it causes an imbalance in the Th1–Th2 ratio, resulting in an exaggerated Th2 immune response, which is associated with the development of allergic conditions. Vitamin B6 enhances immunity in the intestines due to its influence on the formation of the intestinal microbiota. Some studies have revealed that vitamin B6 can alleviate the symptoms and complications associated with SARS-CoV-2 infection, which may be attributed to its ability to suppress the cytokine storm, mitigate oxidative stress, regulate calcium concentration, augment carnosine concentration, and enhance immune function. The findings of research studies indicate that individuals with chronic inflammation exhibit reduced concentrations of vitamin B1. Vitamin B6 enhances immunity in the intestines due to its role in mediating lymphocyte migration into the intestine [129]. In vitro studies have demonstrated the anti-tumor activity of vitamin B6, which inhibits cell proliferation and potentiates the cytotoxicity of chemotherapy in cancer models. The evidence suggests that vitamin B6 deficiency is a risk factor for cancer development. A meta-analysis of studies concluded that an increase of 1 mg/day in vitamin B6 intake corresponded to a 16% reduction in the risk of esophageal cancer. Large prospective cohort studies and randomized controlled trials are needed in order to support their findings and clarify the underlying mechanisms. The effects of an overdose may include nerve damage, numbness in the limbs, or impaired balance [130].

#### 2.3.6. Vitamin B12

Vitamin B12, also known as cobalamin, is a water-soluble vitamin involved in manifold functions within the human body. It takes part in the metabolism of carbohydrates, lipids, and proteins. Moreover, it possesses antioxidant properties. In addition, it is necessary for the synthesis and regulation of DNA. Vitamin B12 plays a crucial role in hematopoiesis, as its deficiency can result in megaloblastic or pernicious anemia [131]. Vitamin B12 is predominantly found in animal-derived foods; therefore, individuals adherent to a vegan diet may be at an elevated risk for deficiency and may necessitate supplementation. The richest sources of cobalamin are liver, meat, dairy products, and eggs.

Vitamin B12 exhibits immunomodulatory properties. It increases the number of NK cells and CD8^+^ T cells, thus boosting the immune response to infection. It serves a regulatory function in the immune response due to its capacity to act as a negative regulator of NF-κB. Furthermore, vitamin B12 interacts with the gut microbiota, which has a significant impact on immune function and supports the gut barrier. Vitamin B12 has antiviral activity. It affects a number of processes pertinent to viral replication, interferon production, phagocyte activity, and T lymphocyte maturation. Consequently, an insufficient concentration of vitamin B12 may elevate the probability and severity of infections. The serum level of vitamin B12 appears to be associated with a positive prognosis in some viral diseases, but most studies on this topic to date have been conducted in patients infected with HIV; therefore, more high-quality studies in patients with other viral conditions are needed [125,131,132]. A severe deficiency of vitamin B12 results in an abnormally elevated CD4^+^/CD8^+^ ratio, diminished NK cell activity, and lymphocyte downregulation. Research findings have shown that intramuscular vitamin B12 injections can normalize the CD4^+^/CD8^+^ ratio and restore NK cell activity in patients with vitamin B12 deficiency [133]. Patients with cancer have been reported to present abnormally high plasma concentrations of vitamin B12, which highlights concerns regarding the safety of vitamin B12 supplementation. According to research findings, elevated plasma vitamin B12 concentrations are associated with a higher risk of liver cancer. However, these results are not specific for cancer, because all disorders which affect the liver can cause high B12 vitamin concentrations. Liver tissue and cell damage is a risk factor for developing liver cancer, and that is why the degree of damage could drive the link between the concentration of vitamin B12 and cancer. For other cancers, the results are inconsistent across the studies. Further studies are needed to clarify the correlation between elevated B12 levels and cancer. According to the current state of knowledge, it is required to treat vitamin B12 deficiencies in patients with cancer who need such treatment [134].

#### 2.3.7. Folic Acid

Folic acid, also referred to as vitamin B9 or simply folate, is a water-soluble vitamin. In the human body, it is essential for manifold cellular pathways, particularly those involved in the production and repair of DNA. Folate is known for its protective properties against neural tube defects, especially spina bifida [133]. The primary dietary sources of folic acid include peanuts, sunflower seeds, asparagus, and lettuce [124].

Folic acid improves both cell-mediated and humoral immunity. Insufficient concentrations of folic acid result in reduced serine levels, since folic acid improves the glycine to serine conversion. Folic acid deficiency can thus preclude the proper formation of antibodies and disrupt the functioning of effector T cells. Consequences of folic acid deficiency on immune functions are associated with abnormalities in the synthesis of DNA and RNA and disruptions in methyl metabolism because these processes are dependent on folate availability [124,134]. In light of the findings from research studies, it can be concluded that folic acid supplementation is associated with a reduction in infection rates, an increase in phagocytosis, an upregulation of immunoglobulin production, and a positive impact on T lymphocyte proliferation. Nonetheless, it appears that folic acid has no *effect* on NK cell activity itself. Additionally, folic acid serves as a survival factor for regulatory T cells, which express high concentrations of the vitamin B9 receptor [124,135]. Folate improves the antiviral and pro-inflammatory molecular pathways of B lymphocytes. A growing body of evidence indicates that low concentrations of folic acid are associated with a more severe course of illness in patients with COVID-19, which highlights the potential therapeutic value of vitamin B9 in managing the disease [123,136]. A deficiency of folic acid affects immune function, resulting in the improper functioning of immune cells, autoimmune responses, disrupted antigen presentation, and inefficient viral clearance. The literature review reveals conflicting evidence regarding the association between folic acid concentration and cancer risk. While some studies have indicated a potential link, other research has suggested that high levels of folic acid may contribute to the growth and progression of existing cancers [128].

Studies suggest that in heavy smokers, high levels of folic acid enhance the cancerogenic effect of smoking. An interesting study was conducted in Poland, which included 132 patients with lung cancer and 396 controls. Among both groups, the median cigarette pack per years of smoking was 30.0. The results showed that an abnormally high folic acid serum concentration (>17.5 nmol/L among the healthy controls) was correlated with a higher risk of lung cancer. The relationship between folic acid and cancer deserves an in-depth evaluation because the safety of supplementation is a key aspect [137]. The optimal time and doses of folic acid supplementation during pregnancy remain controversial. Studies suggested a possible 8.2-fold increased incidence of autism associated with a prenatal supplementation of over 1000 μg of folic acid. Further studies investigating the consequences of folic acid oversupplementation during pregnancy are clearly needed [133].

#### 2.3.8. Other B Vitamins

Vitamin B2 (riboflavin) is a cofactor for many enzymes; hence, it affects several metabolic processes and is eminent for the proper functioning of the immune system. It presents anti-inflammatory, antioxidant, and anti-tumor properties. Vitamin B2 deficiency reduces the ability to conduct an adequate immune response. It also leads to increased oxidative stress. Vitamin B2 boosts macrophage phagocytosis and increases the proliferation of macrophages and neutrophils. Studies conducted on mice indicate that in LPS-induced septic shock, vitamin B2 reduces mortality by decreasing the number of pro-inflammatory cytokines [128].

Vitamin B3, also known as niacin, nicotinic acid, or nicotinamide, serves as a precursor of NAD and NADPH and therefore is important for the activity of sundry enzymes [128]. It also has some immunomodulatory properties. First of all, it boosts the innate immune system. Administered at a high dose, it protects against Staphylococcus aureus infections. Moreover, it reduces inflammation by reducing the concentration of inflammatory factors—IL-6 and TNF-α. There are studies which confirm the beneficial role of niacin in certain types of cancer. A 16-year follow-up study consisting of 650 hepatocellular carcinoma patients showed that higher vitamin B3 intake was associated with a lower risk of this cancer. However, it is still an active field of research because there is a lack of knowledge about the mechanisms through which niacin acts in order to prevent cancer. There are isolated reports which indicate that vitamin B3 in high doses may cause acute hepatitis [128,137,138].

Vitamin B5 (pantothenic acid) is essential for CoA synthesis [128]. It modulates both innate and adaptive immunity. Given the findings of some research papers, it may have a therapeutic effect on patients with tuberculosis as it presents antibacterial and pro-inflammatory properties. Studies have shown that dexpanthenol, a derivative of vitamin B5, reduces oxidative stress in patients with endometriosis and decreases intestinal damage in patients with necrotizing enterocolitis. According to some research results, a higher intake of vitamin B5 may be associated with higher rates of genome damage, which is a biomarker for a higher risk of cancer [128,137,138].

Vitamin B7 (biotin) is essential for human metabolism. It serves as a cofactor for several enzymes which are involved in carboxylation reactions. Vitamin B7 is crucial in the development of chronic inflammation. Some research results indicate that it may have beneficial effects on the treatment of metal allergies [139].

Daily requirements and serum concentrations for known micro- and macroelements and vitamins are presented in Table 1.

**Table 1:** Daily requirements and serum concentrations for known micro- and macroelements and vitamins in adults. Notes: RAE (Retinol Activity Equivalents): A unit used to measure vitamin A. 1 µg RAE = 1 µg retinol = 12 µg beta-carotene. Serum ranges may vary depending on laboratory standards and population. Daily requirements vary depending on age, gender, physiological state (e.g., pregnancy and lactation), and physical activity levels [140,141].

Nutrient	Daily Requirement	Serum Concentration
Macroelements		
Calcium (Ca)	1000–1200 mg	2.2–2.6 mmol/L (8.8–10.4 mg/dL)
Magnesium (Mg)	300–400 mg	0.75–1.0 mmol/L (1.8–2.4 mg/dL)
Potassium (K)	3500–4700 mg	3.5–5.0 mmol/L
Sodium (Na)	1500–2300 mg	135–145 mmol/L
Phosphorus (P)	700 mg	0.81–1.45 mmol/L (2.5–4.5 mg/dL)
Chloride (Cl)	2300 mg	96–106 mmol/L
Microelements		
Iron (Fe)	8–18 mg (pregnant women: 27 mg)	10–30 µmol/L (60–170 µg/dL)
Zinc (Zn)	8–11 mg	10–18 µmol/L (65–110 µg/dL)
Copper (Cu)	0.9–1.2 mg	11–22 µmol/L (70–140 µg/dL)
Selenium (Se)	55 µg	0.89–1.19 µmol/L (70–90 µg/L)
Iodine (I)	150 µg (pregnant women: 220 µg)	100–200 µg/L
Chromium (Cr)	25–35 µg	0.1–0.2 µg/L
Manganese (Mn)	1.8–2.3 mg	4–15 µg/L
Molybdenum (Mo)	45 µg	0.1–0.3 µg/L
Vitamins		
Vitamin A	700–900 µg (RAE)	1.2–2.8 µmol/L (40–80 µg/dL)
Vitamin D	15–20 µg (600–800 IU)	75–125 nmol/L (30–50 ng/mL)
Vitamin E	15 mg (22.4 IU)	12–46 µmol/L
Vitamin K	90–120 µg	0.2–3.2 nmol/L
Vitamin C	75–90 mg	23–85 µmol/L
Vitamin B1 (thiamine)	1.1–1.2 mg	66–200 nmol/L
Vitamin B2 (riboflavin)	1.1–1.3 mg	136–370 nmol/L
Vitamin B3 (niacin)	14–16 mg	0.5–8.45 µmol/L
Vitamin B6 (pyridoxine)	1.3–1.7 mg	20–125 nmol/L
Vitamin B12 (cobalamin)	2.4 µg	148–740 pmol/L
Folic acid	400 µg	7–40 nmol/L
Biotin (vitamin H)	30 µg	200–500 ng/L
Pantothenic acid	5 mg	1–10 µmol/L

### 2.4. Additional Immunomodulatory Compounds

Macro- and micronutrients, along with vitamins, are indisputably among the most crucial compounds for modulating the immune system. It would be remiss not to consider other substances, including those affecting the homeostasis of the gastrointestinal tract, where the largest number of immunocompetent cells are located and are closely connected with other systems of the human body.

#### 2.4.1. Omega-3 Fatty Acids

Omega-3 fatty acids, including α-linolenic acid (ALA), eicosapentaenoic acid (EPA), docosahexaenoic acid (DHA), docosapentaenoic acid (DPA), and stearidonic acid (SDA), are integral to the optimal functioning of the immune system. They contribute to this process in several ways, including modulating the intestinal microflora, reducing inflammation, and regulating the function of immune-competent cells [142,143,144,145].

Studies have demonstrated that omega-3 fatty acids exert a beneficial influence on the intestinal microflora. For instance, supplementation with fish oil rich in EPA and DHA has been shown to increase the abundance of beneficial bacteria such as Bifidobacterium and Lactobacillus in the gut microbiota. In a randomized controlled trial involving 22 healthy individuals, a daily intake of 9.6 g of fish oil for six weeks resulted in significant increases in these beneficial bacterial populations. This modulation of gut microbiota leads to an increase in the production of short-chain fatty acids (SCFAs) with anti-inflammatory properties and a reduction in the formation of pro-inflammatory factors [146,147].

Omega-3 fatty acids also directly affect inflammation in the body. EPA and DHA metabolically compete with arachidonic acid, reducing the formation of its pro-inflammatory metabolites. Consequently, the concentration of pro-inflammatory mediators, including TNF, IL-1β, IL-6, IL-8, PGE2, and leukotrienes series 4, is diminished. In addition, the activity of adhesion molecules such as MCP-1, ICAM-1, VCAM-1, and selectins is reduced, enhancing the anti-inflammatory effect. A meta-analysis of 17 randomized controlled trials, encompassing a total of 823 participants, found that omega-3 supplementation significantly decreased levels of TNF-α and IL-6, indicating a systemic anti-inflammatory effect [145,148,149].

At the cellular level, omega-3 fatty acids integrate into the membranes of immune cells, increasing their fluidity and altering signaling pathways. For example, in macrophages, they lead to changes in gene expression, resulting in less production of pro-inflammatory factors. In a study involving 18 healthy adults, supplementation with 4 g of EPA and DHA daily for 12 weeks led to a significant incorporation of these fatty acids into monocyte membranes, which was associated with a decreased production of pro-inflammatory cytokines upon stimulation. The capacity of omega-3 fatty acids to modify cell membranes and signaling pathways renders them pivotal regulators of the immune response [145,148,149]. In conclusion, omega-3 fatty acids support the immune system through anti-inflammatory effects, the support of gut microflora, and the modulation of immune-competent cell function, making them an extremely important part of a healthy diet [142,143,144,145].

#### 2.4.2. Prebiotics

Prebiotics are food components that are not digested in the gastrointestinal tract but rather stimulate the growth and activity of beneficial microorganisms in the gut, thereby providing health benefits to the host. They are mainly dietary fiber compounds. Prebiotics encompass a range of compounds, including inulin, fructooligosaccharides (FOS), galactooligosaccharides (GOS), arabinoxylans, mannanooligosaccharides (MOS), xylooligosaccharides (XOS), beta-glucans, resistant starch, pectin, polydextrose, lactulose, and soybean oligosaccharides [150,151]. Prebiotics play a significant role in modulating the immune system and influencing host health by interacting with the gut microbiota. Their immunomodulatory properties derive from their capacity to stimulate the growth and activity of beneficial gut bacteria, with Bifidobacterium and Lactobacillus being of particular importance and the subject of extensive study. Prebiotics are fermented by gut microbes, resulting in the production of short-chain fatty acids (SCFAs), such as acetate, propionate, and butyrate, which belong to the group of postbiotics. The effect of prebiotics is the stimulation of gut microbiota, which translates into the immunostimulatory properties of prebiotics, resulting from their ability to stimulate and nourish the microflora. They are used in cases where the restoration and stimulation of the gut microbiome are required, particularly in inflammatory bowel diseases [150,151,152,153,154].

#### 2.4.3. Probiotics

Probiotics are defined as selected strains of bacteria and yeast that, when supplemented, support the health and well-being of the body. They are used in the treatment of various conditions, primarily in inflammatory bowel diseases, but also in allergic, metabolic, and neurodegenerative diseases. The most commonly utilized probiotics encompass Gram-positive bacteria, including Lactobacillus, Bifidobacterium, and Enterococcus. Probiotics also include yeast, with the particularly well-studied species Saccharomyces boulardii being a notable example. Their effects include the restoration of equilibrium within the intestinal microflora, colonization of the gastrointestinal tract, influence upon immune cells, and enhancement of the integrity of the intestinal barrier and mucus production [155,156,157]. The colonization of the gut by probiotics impedes the colonization of the gut by pathogenic bacteria. This occurs as a result of competition for resources and the secretion of compounds that are toxic to harmful microorganisms. Probiotics also interact with pattern recognition receptors (PRRs) present in immune cells, which results in the initiation of signaling cascades that lead to immune cell proliferation and cytokine release. The effects of probiotics can vary depending on the strain of bacteria or yeast involved. However, in most cases, there is an increase in the production of anti-inflammatory cytokines. A significant mechanism of action of probiotics is the activation of B lymphocytes in the intestinal lamina propria. Upon activation, B cells differentiate into plasma cells, which produce IgA antibodies. These antibodies play a pivotal role in the intestinal mucosa’s humoral response, protecting against infections [150,157,158,159,160]. It is also important to note the significance of the metabolites produced by probiotics. They demonstrate anti-inflammatory properties and facilitate intestinal barrier function. The subsequent section will provide a more detailed analysis of these metabolites, which are referred to as postbiotics [155,157]. A preparation that combines probiotics and prebiotics in one product is called synbiotics.

#### 2.4.4. Postbiotics

Postbiotics are defined as substances secreted by probiotic microorganisms that exert beneficial effects on the host body. The definition of postbiotics is not uniform across sources, as some include inactivated probiotics or their lysates within the definition of postbiotics. Postbiotics are particularly suitable for patients with underlying health conditions as they do not contain live cells, which may pose a risk to those with compromised immune systems. The most significant postbiotics encompass SCFAs, tryptophan metabolites, and bacteriocins. If they contain bacterial cell fragments, they exhibit immunomodulatory effects comparable to those observed in probiotics, which are achieved through their interaction with PRR receptors on immune cells [150,157,161].

Short-chain fatty acids are mainly acetic acid (AA), propionic acid (PA), and butyric acid (BA). There is little evidence to suggest that acetic acid provides significant health benefits. In contrast, propionic acid prevents Salmonella infections and reduces the activity of COX enzymes and the proliferation of immune cells, which can result in a decrease in pro-inflammatory cytokines. The action of butyric acid will be discussed in detail in a further subsection [150,162,163]. Bifidocins, which are produced by bacteria of the genus Bifidobacterium, serve as an illustrative example of bacteriocins. These compounds are distinguished by their broad bactericidal activity, which effectively eliminates both Gram-positive and Gram-negative bacteria, as well as certain yeast species [157,163]. Tryptophan metabolites, including indoles and their derivatives, exert a pivotal influence on the regulation of intestinal microflora and intestinal barrier function through the activation of the AhR receptor. They exhibit anti-inflammatory effects, modulating the immune response and assisting in maintaining equilibrium between intestinal health and disease [150,164].

Sodium butyrate is a short-chain fatty acid that is formed in the intestines as a result of the fermentation of dietary fiber by the intestinal microbiota. It is a pivotal metabolite for maintaining the health of the gastrointestinal tract, with both energetic and immunomodulatory functions. Butyrate modulates the inflammatory response in the gut by inhibiting the production of pro-inflammatory cytokines, including TNF-α, IL-6, and IL-1β, while simultaneously increasing the secretion of the anti-inflammatory cytokine IL-10. Moreover, it induces dendritic cell tolerance, which promotes immune tolerance and reduces inflammation [165,166,167]. Furthermore, it has a beneficial effect on the proliferation of differentiated epithelial cells, thereby promoting intestinal homeostasis. Conversely, in cancerous and undifferentiated cells, it has been observed to inhibit their growth, induce cell cycle arrest, and promote apoptosis, a phenomenon which is referred to as the “butyrate paradox” [168]. Butyrate has a major role in inflammatory bowel diseases by reducing inflammation and promoting epithelial regeneration, as well as in the prevention and treatment of colorectal cancer by inhibiting tumor cell proliferation. It enhances the functionality of the intestinal barrier, thereby reducing the translocation of endotoxins and pathogens into the bloodstream, which is a significant factor in the prevention of inflammatory diseases. Additionally, it influences glucose and lipid metabolism, thus facilitating the treatment of metabolic syndrome and type 2 diabetes [169,170].

#### 2.4.5. Alpha-Lipoic Acid

Alpha-lipoic acid is a potent antioxidant. ALA reduces oxidative stress by neutralizing reactive oxygen species (ROS) and regenerating endogenous antioxidants such as glutathione, vitamins C and E, and chelate metals to prevent free radical formation [171,172]. It inhibits the activation of transcription factors associated with inflammation, such as NF-κB, leading to the reduced production of pro-inflammatory cytokines such as interleukin-1β, interleukin-6, and tumor necrosis factor-alpha (TNF-α) [171,172,173]. ALA promotes a shift in the immune response from Th1 (pro-inflammatory) lymphocyte dominance toward Th2 (anti-inflammatory) lymphocytes [172]. The efficacy of this treatment is dependent on the dosage administered, with higher doses demonstrating greater effectiveness and a superior tolerability profile. ALA demonstrates considerable therapeutic potential in conditions associated with inflammation and oxidative stress [174].

#### 2.4.6. Coenzyme Q10

Coenzyme Q10 is a promising nutraceutical that supports the immune system. It exhibits immunostimulating properties. It is a naturally occurring substance within the body that performs antioxidant functions, regulates energy processes at the cellular level, and modulates inflammatory processes by inhibiting or enhancing the activity of cytokines and proteins. As a lipophilic molecule, CoQ10 is naturally found within mitochondrial inner membranes and possesses distinctive antioxidant capabilities [175,176].

Ubiquinone is involved in the process of cellular respiration. This substance is easily reduced to ubiquinol and transfers electrons from complexes I and II to cytochrome B, so it is directly involved in ATP synthesis. Through the ability to cycle between redox states, ubiquinone is essential for the efficient execution of cellular respiration, as well as for neutralizing the free radicals that are formed during this process. Its deficiency leads to a decrease in ATP and increased oxidative stress, which can lead to the development of metabolic and degenerative diseases [175,177].

Coenzyme Q10 supplementation results in a decrease in the concentration of interleukin-6 (IL-6), which acts as a pro-inflammatory cytokine in conjunction with IL-1β and TNF-α. This cytokine is involved in the development of autoimmune diseases and chronic inflammation [175]. Its immunomodulatory properties also include the ability to increase the activity of antioxidant enzymes and diminish reactive oxygen species, which are not only detrimental to immune cells but also induce the expression of genes responsible for the development of inflammation [175,176,178]. Moreover, it facilitates immune equilibrium by enhancing the activity of anti-inflammatory cytokines, such as IL-10 [172]. Additionally, it enhances the function of T lymphocytes, particularly by protecting their mitochondria from oxidative damage, resulting in a more efficient immune response. Its key role in cellular energetics promotes immune cell activation [176,177,179]. It also acts on macrophage levels, reducing their activation toward an M1 (pro-inflammatory) profile [177,180]. Coenzyme Q10 regulates the activity of the complement system, crucial in defense against pathogens, while protecting against its excessive activity, leading to tissue damage. It also supports the regeneration and proliferation processes of immune system cells, such as B lymphocytes, responsible for antibody production. It affects signaling pathways involved in lymphocyte proliferation and differentiation, such as NF-κB and PI3K/Akt, which control immune cell survival and activity [175,177,178]. Under conditions of metabolic stress, the body’s cells, including those of the immune system, enter the apoptosis pathway. Ubiquinone counteracts this process by stabilizing the mitochondrial membrane potential and reducing signals leading to cell death [180,181]. Consequently, coenzyme Q10 facilitates the modulation of both innate and acquired immunity, rendering it a prospective therapeutic adjunct in the management of autoimmune disorders, infections, and chronic inflammation.

#### 2.4.7. Plant Compounds and Their Derivatives

Plants have long been valued for their rich composition of bioactive compounds that support health and well-being. Many of them are natural sources of many valuable compounds, such as phenolic acids, flavonoids, coumarins, alkaloids, polyacetylenes, saponins, carotenoids, terpenoids, and menthol. The presence of these compounds not only provides antioxidant and anti-inflammatory effects but also supports various physiological processes.

##### Alkaloids

Alkaloids are a group of natural chemical compounds with significant pharmacological properties, playing a crucial role in the prevention and treatment of various human diseases. Their effects are mediated through interactions with specific molecules, such as nuclear factor-κB and cyclooxygenase-2, or by modulating acetylcholinesterase activity, influencing processes like inflammation, oxidative stress, and immune responses. Studies have shown that alkaloids exhibit protective effects in cardiovascular, cancer, and neurodegenerative diseases due to their antioxidant, anti-inflammatory, and anticancer properties [182].

For instance, berberine, an alkaloid found in plants like Rhizoma Coptidis, has been linked to lowering blood glucose concentration and improving cardiovascular health [183]. Similarly, curcumin, an alkaloid in turmeric, is well known for its anti-inflammatory properties and potential neuroprotective effects [184,185]. The broad biological activity of alkaloids underscores their potential to reduce disease risk and promote overall health.

##### Phenolic Acids

Phenolic acids are a group of chemical compounds widely found in plants. They exhibit potent antioxidant activity, neutralizing free radicals and preventing oxidative-stress-induced cellular damage. Additionally, they possess anti-inflammatory, anticancer, and cardioprotective properties, making them the focus of research for preventing conditions such as heart disease, diabetes, and cancer [186,187,188].

The protective effects of phenolic acids are primarily attributed to their ability to inhibit pro-inflammatory enzymes (e.g., cyclooxygenase) and modulate lipid metabolism by reducing lipid peroxidation [189]. Research also highlights their potential to improve gut microbiota, enhance digestive system function, and strengthen immune responses [190]. Phenolic acids are present in various plant-based foods, including coffee, tea, fruits, vegetables (e.g., carrots and purple potatoes), cereals, and seeds [191,192,193].

##### Coumarins

Coumarins are a group of chemical compounds found in numerous plants, recognized for their significant potential in disease prevention due to their diverse biological properties. They are extensively studied for their anti-inflammatory, antioxidant, anticancer, anticoagulant, and antimicrobial effects [194].

Coumarins such as warfarin and acenocoumarol are well-known anticoagulants. As vitamin K antagonists, they inhibit enzymes responsible for synthesizing clotting factors in the liver, reducing thrombus formation [195,196].

Coumarins exhibit anticancer potential by inducing apoptosis in cancer cells and exerting cytotoxic effects [194,197]. For instance, umbelliferone has demonstrated the ability to inhibit cancer cell growth in various tumor models [198]. Their antioxidant activity also plays a key role in protecting DNA from oxidative-stress-induced damage, potentially preventing cancer initiation [199].

Moreover, coumarins can act neuroprotectively by neutralizing free radicals and modulating inflammation in the nervous system [200,201]. They also possess antimicrobial properties, making them potential agents for preventing bacterial, viral, and fungal infections [202]. For example, osthole, a coumarin found in certain plants, exhibits potent antibacterial activity against antibiotic-resistant strains [203].

##### Polyacetylenes

Polyacetylenes are bioactive compounds in plants such as carrots, celery, and marigolds. They exhibit anticancer, anti-inflammatory, antibacterial, and antifungal properties [204,205]. For example, falcarinol and falcarindiol, found in carrots, reduce the risk of colorectal cancer in mouse models by inhibiting cancer cell proliferation and inducing apoptosis, offering potential therapeutic applications in humans [206].

Due to their antioxidant and anti-inflammatory properties, polyacetylenes support cardiovascular health and may act neuroprotectively, making them promising for preventing Alzheimer’s disease [207]. They also support glucose metabolism, suggesting potential applications in preventing and treating type 2 diabetes [208]. While polyacetylenes hold therapeutic promise, further research is needed to establish their safety and optimal dosing.

##### Saponins

Saponins are natural compounds in many plants, such as soy, alfalfa, beans, ginseng, and horse chestnut. Their name derives from their ability to form foam in aqueous solutions, attributed to their structure as glycosides with a sugar moiety and an aglycone (sapogenin) [209,210]. Saponins exhibit various health-promoting activities, making them valuable in disease prevention.

Saponins can inhibit cancer cell growth, induce apoptosis, and suppress angiogenesis [211,212]. For instance, saponins from ginseng have demonstrated efficacy in models of colorectal and breast cancer [213,214].

Through immune system modulation, saponins enhance immune responses and alleviate chronic inflammation, a key factor in many chronic diseases [215]. They also display antibacterial, antifungal, and antiviral effects, such as inhibiting pathogenic bacteria like Helicobacter pylori and reducing the risk of fungal infections [216,217].

##### Terpenoids

Terpenoids, or isoprenoids, are diverse natural compounds found in plants, algae, and certain microorganisms. Derived from isoprene units, they exhibit varied structures and a broad spectrum of health-promoting effects, making them crucial in disease prevention. Prominent terpenoids include carotenoids (e.g., beta-carotene), monoterpenes (e.g., limonene), and sesquiterpenes (e.g., artemisinin) [218,219,220].

Beta-carotene and limonene can inhibit cancer cell growth and induce apoptosis [221,222]. Terpenoids like artemisinin from Artemisia annua are being studied as potential anticancer agents [223,224]. Carotenoids, such as lycopene found in tomatoes and watermelons, may reduce the risk of heart disease through anti-inflammatory effects and oxidative stress reduction [225,226].

Some terpenoids, such as ginkgolides from Ginkgo biloba, improve cognitive function, act neuroprotectively, and may prevent Alzheimer’s disease [227,228,229,230]. Sesquiterpenes like artemisinin exhibit strong antimicrobial properties, combating bacteria, viruses, fungi, and protozoa.

Menthol, a monoterpene from peppermint, exerts cooling and analgesic effects by activating cold receptors (TRPM8) [231,232]. It relieves muscle pain, nasal congestion, respiratory infections, and digestive discomforts like bloating and irritable bowel syndrome [232,233,234,235,236]. Menthol exhibits anti-inflammatory, smooth muscle relaxant, and antimicrobial properties [231,237].

## 3. Conclusions

Optimal nutrition entails not only the intake of adequate amounts of macronutrients and micronutrients but also the consideration of the role of non-nutritive bioactive compounds that can modulate the functioning of the immune system and affect the overall condition of the body. The information presented in this article indicates that not only macronutrients, micronutrients, and vitamins, but also omega-3 fatty acids, probiotics, prebiotics, postbiotics, coenzyme Q10, sodium butyrate, and lipoic acid make a considerable contribution to anti-inflammatory, regenerative, and immunomodulatory processes.

A summary of the effects of non-nutritive bioactive compounds on the immune system is presented in Table 2.

Of particular importance is a comprehension of the synergistic action of these components in maintaining the body’s homeostasis. An understanding of this action may lead to new avenues of research in the prevention and treatment of diseases that are associated with immune dysfunctions.

Emphasizing an interdisciplinary approach to exploring the interactions between nutrition and immunity is fundamental to developing innovative strategies to promote health. The findings presented in this article indicate a need for further research to more fully elucidate the potential of bioactive components in the context of optimal nutrition and preventive medicine.

## Figures and Tables

**Table 2:** A summary of the effects of non-nutritive bioactive compounds on the immune system [39,77,78,79,80,84,119,150,170,182,183,184,185,186,187,188,189,190,191,192,193,194,195,196,197,198,199,200,201,202,203,204,205,206,207,208,209,210,211,212,213,214,215,216,217,218,219,220,221,222,223,224,225,226,227,228,229,230,231,232,233,234,235,236,237].

Immune Function Roles	Non-Nutritive Bioactive Compounds
Maintaining the structural and functional integrity of innate barriers (e.g., skin, respiratory tract, and digestive tract)	Vitamins: A, D, E, C, B6, B12, folate; iron, zinc, omega-3 fatty acids, probiotics, prebiotics, sodium butyrate, phenolic acids
Differentiation, proliferation, functioning, and movement of innate immune cells	Vitamins: A, D, E, C, B6, B12, folate; zinc, iron, copper, selenium, magnesium, calcium, sodium, iodine, manganese
Roles in inflammation, antioxidant effects, and effects in oxidative burst	Vitamins: A, D, E, C, B6; zinc, iron, copper, selenium, magnesium, potassium, iodine, cobalt, chromium, manganese, omega-3 fatty acids, probiotics, prebiotics, sodium butyrate, coenzyme Q10, alkaloids, phenolic acids, coumarins, polyacetylenes, saponins, terpenoids
Differentiation, proliferation, and normal functioning of T cells	Vitamins: A, D, C, E, B6, B12, folate; zinc, iron, copper, selenium
Antibody production and development	Vitamins: A, D, C, E, B6, B12; folate, zinc, copper, selenium, magnesium, probiotics, prebiotics
Responses to antigen	Vitamins: A, D, E, folate, zinc, magnesium
Activity of the complement system	Vitamins: A, D, E, C, B6, B12, folate; iron, zinc, selenium, magnesium, probiotics, prebiotics, coenzyme Q10
Antimicrobial effects	Vitamins: A, D, C; zinc, iron, copper, selenium, probiotics, prebiotics, coenzyme Q10, manganese, coumarins, polyacetylenes, saponins, terpenoids

## Data Availability

No new data were created or analyzed in this study. Data sharing is not applicable to this article.
